# Electrostatic MEMS Vibration Energy Harvesters inside of Tire Treads

**DOI:** 10.3390/s19040890

**Published:** 2019-02-21

**Authors:** Yasuyuki Naito, Keisuke Uenishi

**Affiliations:** 1 Institute for Energy and Material Food Resources, Technology Innovation Division, Panasonic Corporation, Kyoto 619-0237, Japan; 2 Department of Management of Industry and Technology, Graduate of School of Engineering, Osaka University, Osaka 561-0871, Japan; uenishi@mit.eng.osaka-u.ac.jp

**Keywords:** power MEMS, vibration energy harvester, electret electrostatic, TPMS, tire sensor

## Abstract

An electret electrostatic MEMS vibration energy harvester for tire sensors mounted inside of the tire tread is reported. The device was designed so as to linearly change an electrostatic capacitance between the corrugated electret and output electrode according to the displacement of the proof mass. The electromechanical linearity was effective at reducing the power loss. The output power reached 495 μW under sinusoidal vibration despite the footprint size being as small as 1 cm^2^. Under impact vibration inside of the tire tread, the output power reached 60 μW at a traveling speed of 60 km/h. It was revealed that a higher mechanical resonance frequency of the harvester adjusted within the frequency band of a low-power spectral density of impact vibration acceleration was effective for high efficiency harvest impact vibration energy.

## 1. Introduction

In order to improve the driving safety of automobiles, the requirements of tire pressure monitoring systems (TPMS) have advanced worldwide, starting with the United States and Europe. A tire sensor with the TPMS inside of tire tread enables gathering information of the road surface and tires for the detection of the degree of slippage. It is possible to control the attitude of the vehicle safely and comfortably. The tire sensor consists of not only a pressure sensor, but also an acceleration sensor and a temperature sensor, which improves the detection accuracy by analyzing the multifaceted data at a high frequency. It is necessary to supply stable power to the tire sensor during the life of tire because replacement of the power supply is not acceptable in consideration of user convenience and the reduction of cost.

As a power source, the battery capacity is insufficient to be applied here. For this reason, we tried to apply environmental power harvesters, which convert energy in the environment into electric energy. The environmental power harvesters include photovoltaic and thermoelectric types, but they are inappropriate because there is no light source or heat source inside of a tire. On the other hand, a vibration energy harvester could recover the power demand of the tire sensor since vibration energy is abundant inside of the tire [1,2].

An electrostatic vibration energy harvester is one of the expected candidates in power generation methods because the power output density of the piezoelectric type or electromagnetic type is relatively low, and the device size becomes large. The electrostatic type needs an external voltage source to apply potential on electrodes for electric field generation [3]. An electrical charge holding an electret can be used as an internal voltage source of an electrostatic type. The electret vibration energy harvesters are classified mainly as the in-plane overlapping type [4] and the out-of-plane gap-closing type [5]. In the former type, the electrets displace in parallel to output electrodes in-plane. Methods for enhancement of accumulated charges on the output electrodes are reported: a higher electret potential using polymer electret [6], an inorganic insulator SiO_2_/Si_3_N_4_-laminated film in a cost-effective CMOS (Complementary Metal Oxide Semiconductor)/MEMS (Micro Electro Mechanical Systems) production line [7], an increased dielectric constant using ferroelectrics [8] and liquid crystals [9], an opposite-charged electret [10], and a potassium ion electret [11]. Structural optimization is also reported, a smaller air gap between electrets and output electrodes [12], and a mechanical nonlinearity with a spring stopper [13,14]. In most of these works, vibration energy harvesters are evaluated under sinusoidal vibration, which rarely exists in the real environment from the application point of view. Stochastic noise-like vibration tests are reported in the experimental environment [15,16].

For the TPMS and the tire sensors, electret vibration energy harvesters have to be evaluated considering the real impact vibration inside of the tire tread. The previously reported output power was 4.5 μW in the case of Power Spectral Density (PSD) of impact vibration acceleration of 7.3 × 10^−2^ g^2^·Hz^−1^ at a traveling speed of 50 km/h, and a mechanical resonance frequency of the harvester of 550 Hz [2]. In this paper, an order of magnitude higher output power in spite of an order of magnitude lower PSD is reported. It is revealed that the higher resonance frequency of the harvester adjusted within the frequency band of low PSD is effective for high efficiency harvest impact vibration energy. Output power from the harvester of a resonance frequency of 1.2 kHz reached 60 μW in the case of PSD 5.0 × 10^−3^ g^2^·Hz^−1^ at a traveling speed of 60 km/h.

## 2. Design

### 2.1. Basic Concept of an Electrostatic MEMS Vibration Energy Harvester

**Figure 1:**
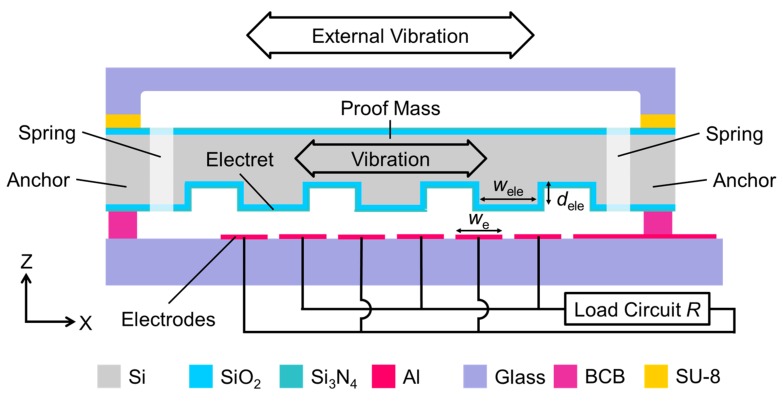
Schematic cross-sectional view of electrostatic MEMS vibration energy harvester. BCB, Benzocyclobutene.

A schematic cross-sectional view of the electret vibration energy harvester is shown in Figure 1. Three substrates are stacked and bonded together. A mechanical resonator is provided on the intermediate substrate, and the proof mass and spring are collectively formed by etching the Si substrate. A stacked film of SiO_2_/Si_3_N_4_ as an electret material is deposited on the corrugated structure of the proof mass and charged to a predetermined potential by a corona discharger. Al output electrodes are made on the lower substrate, which is used to extract generated electricity.

A design concept of the Si spring is the adjustment to a higher mechanical resonance frequency within the frequency band of PSD of the impact vibration acceleration inside of the tire tread, which enables harvesting more power at a high frequency. The PSD in the tangential direction X of a circular tire is low from 5.0 × 10^−3^ g^2^·Hz^−1^–1.0 × 10^−2^ g^2^·Hz^−1^ at 250 Hz–1.5 kHz in the case of a traveling speed of 60 km/h [7]. The resonance frequency of the Si spring is adjusted to a relatively high frequency at 1.2 kHz, which is also an advantage to make the Si spring small and rigid against the impact vibration.

The Si proof mass moves because of resonance under external vibration (sinusoidal vibration or impact vibration). Electric fields formed by charges stored in the electret induce charges at the facing output electrodes [17]. The proof mass displaces parallel to the direction in which strip-shaped output electrodes are arranged. The amount of charges on the output electrode is increased or decreased in response to the change of the electric fields. As a result, alternating current flows in the load circuit connected between the two output electrodes.

### 2.2. Electrode Configuration for High Power Generation

The convex portion of the electret is arranged to face the two output electrodes with the same overlap area. When the proof mass is displaced by the external vibration, the overlap area of the two opposed portions changes, keeping the total opposed area the same. For example, when the area of one facing part increases with a certain value, the area of the other facing part decreases with the same value. The maximum displacement of the proof mass is 100 μm according to the expansion and contraction of the Si spring. A width of the convex portion of the electret is *w*_ele_ = 250 μm. In this case, the output electrode width is designed to be *w*_e_ = 200 μm so that the convex portion displaces within the two opposed output electrodes. Even when the displacement reaches the maximum value, the configuration is designed so as not to displace up to the third output electrode. An electrostatic capacitance between the electret and the one side output electrode changes linearly with respect to the relative displacement. An output signal waveform can be made into a regular sinusoidal wave in order to reduce power loss with the electromechanical linearity [18,19]. A glass insulator is used as the lower substrate for the reduction of parasitic capacitance between the output electrodes and the substrate, which is also effective to increase the power generation, avoiding signal transmission loss.

### 2.3. Electret Structure for a High Charge Ratio on Output Electrodes

The corrugated electret structure is one of features of this developed generator. Conventionally, the electret material is patterned on a flat surface in order to ensure an electric potential difference on the output electrode between the state in which the electret and the output electrode are opposed and the state in which they are not opposed to each other [20]. The electric field of the patterned electret is quite homogeneous due to a fringing field, and it is the cause of the degradation of the electric potential difference between narrow rectangular electrets and spaces. The electret material is deposited on the corrugated structure without the necessity of the electret patterning. A capacitance change can be obtained since the distance from the convex portion or the concave portion to the output electrode varies. It is possible to change the amount of induced charges on the output electrodes and to obtain high power generation capability with this configuration. It is supposed as a note that the non-uniform fringing electric fields of the corrugated electret also have an influence on the movement of the proof mass, even for a submicron irregular displacement, which is linked to energy damping [21].

## 3. Fabrication

### 3.1. Process Flow

**Figure 2:**
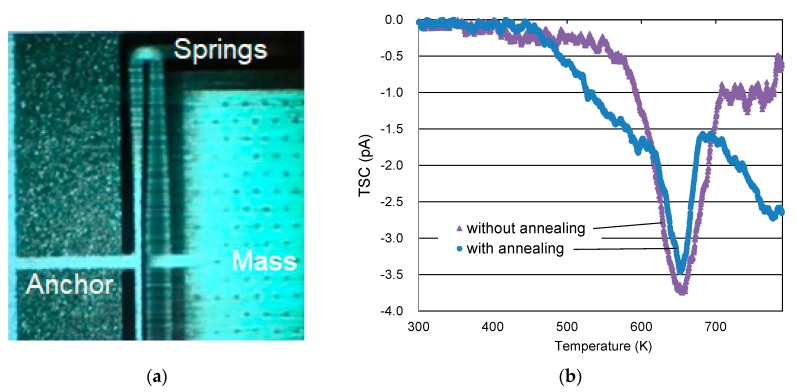
Fabricated electrostatic MEMS vibration energy harvester: (**a**) optical microscope image of the proof mass and spring during vibration; (**b**) Thermally-Stimulated current (TSC) spectrum of the electret SiO_2_/Si_3_N_4_ stacked film.

Firstly, the surface of the middle Si substrate was deeply etched *d*_ele_ = 100 μm by DRIE (Deep Reactive Ion Etching) to form a corrugated structure. Then, 1 μm-thick SiO_2_ and 150 nm-thick Si_3_N_4_ as the electret materials were grown covering the corrugated structure. The proof mass and the springs were formed by DRIE through the whole thickness of 650 μm of the substrate. The SiO_2_/Si_3_N_4_ stacked film was used as the hard mask for the DRIE. The Si spring has a high aspect ratio in the thickness direction. The electret side of the substrate was discharged by a corona discharger to functionalize as an electret. An optical microscope image of the proof mass and the spring during vibration is shown in Figure 2a.

A Al electrode was formed on a glass substrate as the lower substrate, which was bonded to the middle substrate via BCB (Benzocyclobutene). The distance between the electret and the output electrode was adjusted to 10 μm with a thickness of the BCB bonding frame patterned using photolithography. The bonding resin (SU-8) was formed by roller coating on the top glass substrate that was partially engraved, and the top substrate was bonded to and packaged with the middle substrate via the SU-8.

### 3.2. Annealing Process for a Highly-Stable Inorganic Electret

In order to improve the charge retention stability of the electret, heat treatment (annealing) was performed after deposition of SiO_2_/Si_3_N_4_ materials. The charge retention stability was evaluated introducing Thermally-Stimulated current (TSC). The TSC temperature dependence of the inorganic electret is shown in Figure 2b. The TSC’s sharper peak with respect to temperature means higher charge retention stability because higher activation energy was necessary to move the trapped charge. The peak became steep by applying the annealing, the peak width in temperature at TSC −2.0 pA was Δ50 K from 620–670 K with annealing and Δ70 K from 620–690 K without annealing. It was confirmed that the deep potential wells around 650 K for the stable charge trapping were more uniformly formed.

## 4. Measurement Result

### 4.1. Sign-Wave Vibration

**Figure 3:**
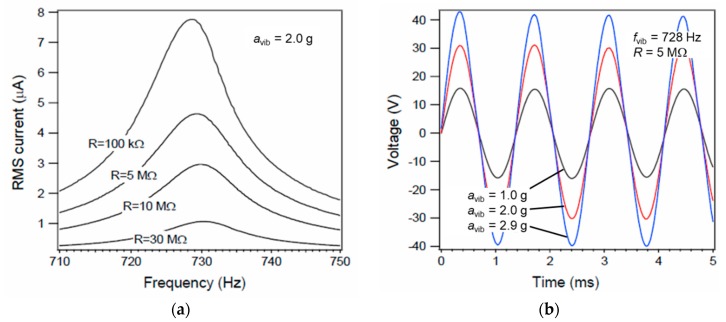
Evaluation of the fabricated electrostatic MEMS vibration energy harvester: (**a**) frequency response; (**b**) output voltage of vibration frequency of 728 Hz, electret potential of 120 V, and road resistance of 5 MΩ.

The basic performance of the fabricated energy harvesters was evaluated under sinusoidal vibration. The frequency response and output voltage waveform are shown in Figure 3a,b. The Root Mean Squared (RMS) value of the output current *i*_rms_ had a Lorentzian shape and good linearity with respect to the frequency of external vibration. The proof mass, the mechanical resonant frequency of the first mode, and the electret potential were *m* = 160 mg, *f*_vib_ = 728 Hz, and *V*_ele_ = 120 V, respectively. The frequency of the external vibration was adjusted to the mechanical resonant frequency, and the acceleration of the external vibration was changed. For the output current *i*_rms_ flowing through load resistance *R*, the average output power *P* can be calculated with the relation of *P* = *R* * *i*_rms_^2^. The load matching was achieved at the load resistance *R* = 5 MΩ. The output voltage amplitude varied linearly with the vibration acceleration. The output voltage amplitude reached the maximum value of 41 V when the proof mass reached the maximum displacement with the vibration acceleration *a*_vib_ = 2.9 g.

**Figure 4:**
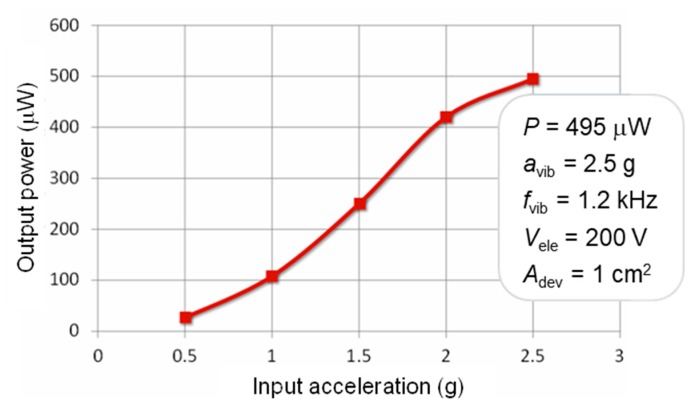
Output power of the fabricated electrostatic MEMS vibration energy harvester of a vibration frequency of 1.2 kHz and an electret potential of 200 V.

On the fabricated energy harvesters, the parameters were increased to the ideal mechanical resonance frequency *f*_vib_ = 1.2 kHz and *V*_ele_ = 200 V. The vibration acceleration dependence of the output power is shown in Figure 4. The high output power of *P* = 495 μW was obtained although the footprint size was as small as about *A*_dev_ = 1 cm^2^.

### 4.2. Impact Vibration in Tire Tread

In order to detect information from the road surface more accurately, a tire sensor was mounted inside of the tire tread. The performances of the fabricated energy harvesters had to be evaluated under impact vibration generated when the tire sensor contacted the road surface. In situ measurements were performed by mounting the energy harvester and an accelerometer inside of the tire tread using an adhesive agent. The acceleration sensor measured the vibration in the tangential direction X, the lateral direction Y, and the radial direction Z of the circular tire. We attempted to harvest the vibration energy in the tire rotation direction X by aligning the vibration direction of the proof mass of the energy harvester.

**Figure 5:**
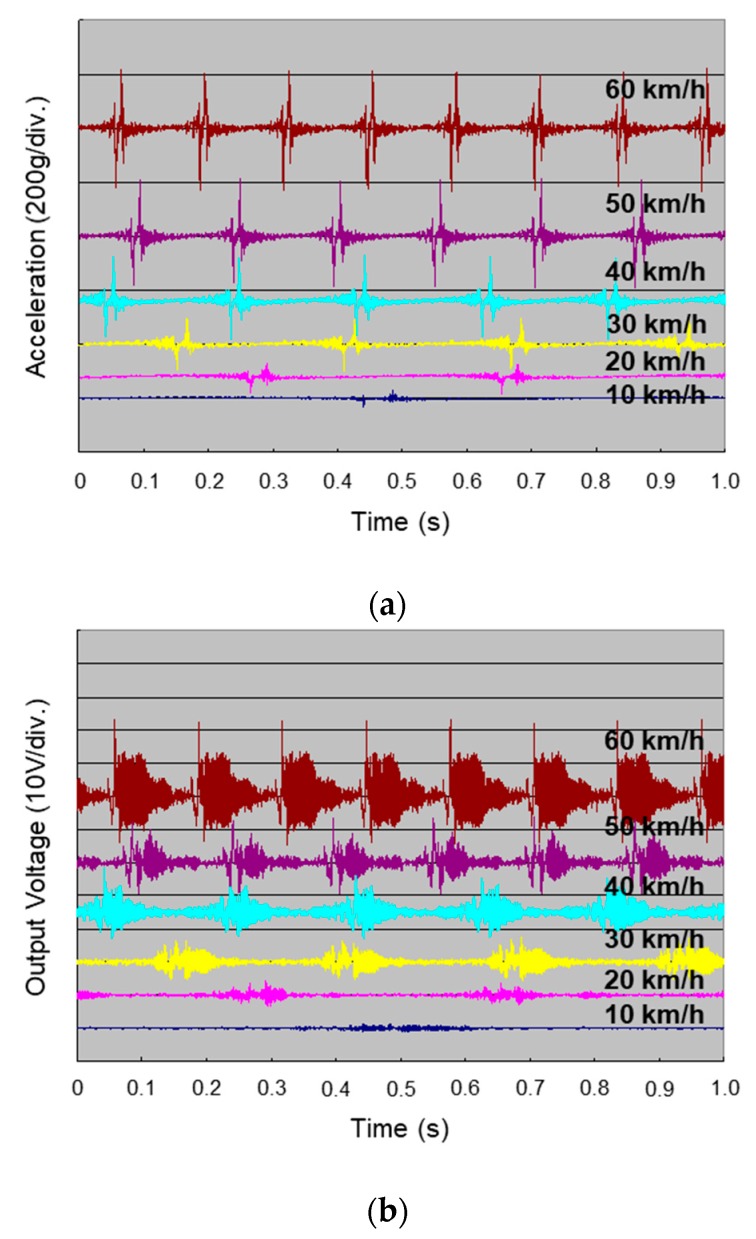
Evaluation of the electrostatic MEMS vibration energy harvester inside of the tire tread: (**a**) impact vibration acceleration; (**b**) output voltage.

**Figure 6:**
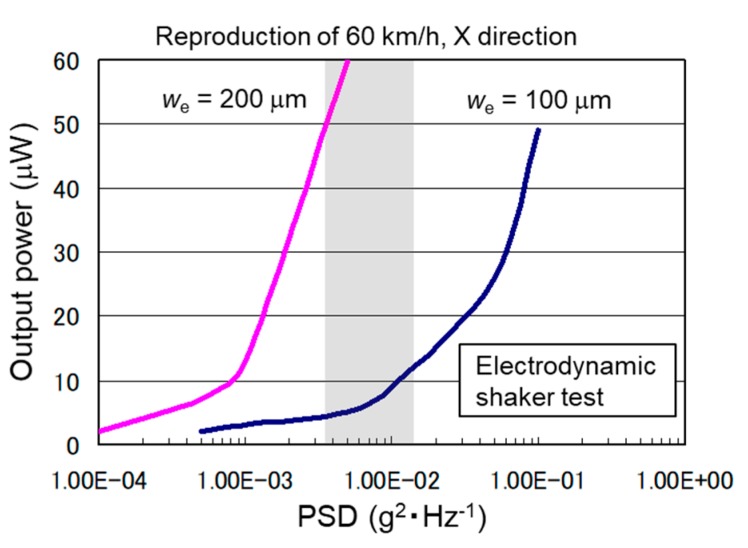
Output power of the electrostatic MEMS vibration energy harvester depending on the Power Spectral Density (PSD) of impact vibration acceleration inside of the tire tread.

The measured acceleration of the impact vibration is shown in Figure 5a. The impact vibration occurred every time the tire rotated, and the acceleration increased at faster traveling speeds. The output voltage of the energy harvester with the impact vibration is shown in Figure 5b. The voltage value became the maximum when the harvester contacted the road surface and then decreased due to free damped oscillation of the proof mass while the harvester detached from the road surface. It was confirmed that the output voltage increased at higher traveling speed. The PSD dependence of the output power is shown in Figure 6. The PSD of the impact vibration acceleration was reproduced by an electrodynamic shaker. The output power 60 μW was obtained in spite of the low PSD value of 5.0 × 10^−3^ g^2^·Hz^−1^ reproduced for a traveling speed of 60 km/h. It was revealed that a higher resonance frequency of spring adjusted to the frequency band of low PSD was effective for high efficiency harvest impact vibration energy.

In the case of the output electrode width *w*_e_ = 100 μm, a capacitance between the electret and the one side output electrode changed non-linearly with respect to relative displacement because the convex portion of the electret reached the next nearest neighbor output electrode when the proof mass reached the maximum displacement. The output wave became an irregular waveform rather than a sinusoidal waveform, and the output power degraded as described in Section 2.2. It was confirmed that the electromechanical linearity was effective to increase the power generation.

The Alternating Current (AC) generated from the vibration energy harvester was converted to Direct Current (DC) using an AC/DC converter in order to use the energy harvester as a DC power supply. We developed a high efficiency AC/DC converter to deal with the high output voltage of the electrostatic generator. The MPPT (Maximum Power Point Tracking) technology was introduced to realize a high conversion efficiency of 88% [22]. In addition, this is a concept of self-starting by generated power, which can eliminate an activating circuit. The vibration energy harvester could be used as a DC power supply for tire sensors.

## 5. Conclusions

We developed vibration energy harvesters inside of a tire tread. An inorganic insulator SiO_2_/Si_3_N_4_ film usable in a Si-based CMOS/MEMS production line was used as an electret material. By making the electret into a corrugated structure, a capacitance change could be obtained since the distance from the convex portion or the concave portion to the output electrode varied. It was possible to change the amount of induced charges on the output electrodes. In addition, the device was designed to change the capacitance between the convex portion of the electret and the output electrode linearly with displacement of the proof mass. The electromechanical linearity was effective at reducing the power loss. The output power reached 495 μW under sinusoidal vibration at an adjusted high frequency 1.2 kHz despite the footprint size being as small as 1 cm^2^.

Under impact vibration inside of the tire tread, the output power reached 60 μW in the case of PSD 5.0 × 10^−3^ g^2^·Hz^−1^ at a traveling speed of 60 km/h. The performance was an order of magnitude higher output power in spite of an order of magnitude lower PSD compared to previous works. It was revealed that the higher mechanical resonance frequency of the harvester adjusted within the frequency band of low PSD was effective for high efficiency harvest impact vibration energy inside of the tire tread.

In further work, it is expected that electrostatic MEMS vibration energy harvesters inside of tire treads will enhance the function of tire sensors, which will enable the evolution of intelligent automobiles.
